# New principles and new paths needed for online research in mental health: Commentary on Burnette et al. (2021)

**DOI:** 10.1002/eat.23670

**Published:** 2022-01-10

**Authors:** Kelly R. Donegan, Claire M. Gillan

**Affiliations:** ^1^ School of Psychology Trinity College Dublin Dublin Ireland; ^2^ Trinity College Institute of Neuroscience Trinity College Dublin Dublin Ireland; ^3^ Global Brain Health Institute Trinity College Dublin Dublin Ireland

**Keywords:** Amazon Mechanical Turk, citizen science, data quality, metascience, online research methods

## Abstract

Online methods have become a powerful research tool, allowing us to conduct well‐powered studies, to explore and replicate effects, and to recruit often rare and diverse samples. However, concerns about the validity and reliability of the data collected from some platforms have reached crescendo. In this issue, Burnette et al. (2021) describe how commonly employed protective measures such as captchas, response consistency requirements, and attention checks may no longer be sufficient to ensure high‐quality data in survey‐based studies on Amazon's Mechanical Turk. We echo and elaborate on these concerns, but believe that although imperfect, online research will continue to be incredibly important in driving progress in mental health science. Not all platforms or populations are well suited to every research question and so we posit that the future of online research will be much more varied, and in no small part supported by citizen scientists and those with lived experience. Whatever the medium, researchers cannot stand still; we must continuously reflect and adapt to technological advances, demographics, and motivational shifts of our participants. Online research is difficult but worthwhile.

## 1 INTRODUCTION

Burnette et al. (2021) present a series of concerns about the quality of data gathered using Amazon Mechanical Turk (AMT). Data come from a study they conducted aiming to characterize eating disorder symptoms in a representative sample of transgender individuals. Their observations regarding data reliability and validity are disturbing and their conclusions compelling. Indeed, in just the past month we have had a remarkably similar experience with a questionnaire‐based study and made the difficult decision that an entire dataset from over 500 AMT participants was of insufficient quality to draw inferences. These experiences are not rare. There is a growing body of literature highlighting that in recent years the rates of usable, good‐quality data collected through AMT have deteriorated, calling into question the continued use of AMT as a recruitment source for research in psychology (Chmielewski & Kucker, 2019).

In this commentary, we echo Burnette et al.'s (2021) concerns with the use of AMT, but aim to add some nuance to that perspective and suggestions for how online research can continue fruitfully, albeit cautiously. Online research has had a transformative impact on research in the brain sciences, allowing us to dramatically scale up studies, increasing statistical power and promoting reproducibility in research through explicitly “exploration and replication” style methods (Gillan & Rutledge, 2021). When working correctly, it helps us diversify samples, overcoming long‐standing biases toward White, wealthy, western, and educated populations. It also facilitates participation of individuals not normally engaged in research, such as those living far from major research centers, those with mobility issues, caring responsibilities or other factors that prevent travel. While the bubble of AMT may have burst, there remain a range of online methods that can be employed outside of this platform to deliver reliable and valid data for psychology researchers. Moreover, we suggest that good quality data can likely still be gathered on AMT for certain study designs (e.g., task‐based studies that incorporate comprehension checks). Regardless of platform, if we are to continue to do research via the Internet, as a field we need to develop a better understanding of the incentives that drive research participation and tailor our research so that the needs of both experimenter and participant can be met. Ultimately, no method is a panacea; when working in the online space, we must constantly adapt as the technology, demographics, interests, and needs of the populations we study evolves over time. This presents new challenges for researchers, but the unique opportunities it affords makes this effort worthwhile.

## 2 WHAT HAPPENED IN BURNETTE ET AL.?

With a carefully designed study, accompanied by a series of reasonable post hoc data integrity checks, Burnette et al. (2021) had clear grounds to exclude 91% of their sample of >3,000 consenting participants. This was done on the basis of not just attention checks and response times, but egregious inconsistencies in their self‐reported data. The most shocking statistics perhaps that their sample dropped in half (*N* = 2,413 to *N* = 1,240) based on inconsistent reporting of their own age and gender‐identity. Although care was taken not to reveal inclusion criteria and to block IP addresses that had already completed the prescreening, the requirement that participants identify as transgender to enter the study likely played a role. Burnette et al. (2021) observed rates of transgender identification of 16% at the prescreening stage, where they note the proportion of the U.S. population identifying as transgender is just 0.6%. This suggests there was some form of leakage of inclusion criteria, be it ineligible participants altering IP addresses and changing their response, or through communication between workers on AMT about entry requirements. Solutions have been proposed to this sort of problem, such as accepting and paying all participants who complete a prescreening survey and inviting only those eligible to the next stage. These are more costly approaches but are viewed as being more fair by workers on the platform (Brawley & Pury, 2016), and have the benefit of not inadvertently incentivizing fraudulent responses. The inclusion criteria, in combination with the low expected base rate of transgender identification, may have had the unintended consequence of enriching the sample for disingenuous respondents. While there are some lessons to be learned here, even with the addition of costly design features it is hard to imagine the proportion of those meeting inclusion criteria rising from the 9% observed to anywhere near an acceptable level.

## 3 NOT JUST NOISE—SYSTEMATIC BIAS AND SPURIOUS CORRELATION

When we think about “bad data” from online platforms, we have formerly excused the issues as being nonsystematic, adding noise that can be compensated for through larger samples (Gillan & Rutledge, 2021). But recent studies have started to question this thinking. Discouragingly, the inclusion of these fraudulent respondents can suppress, inflate or even reverse the sign of observed relationships with cognitive abilities (Zorowitz, Niv, & Bennett, 2021). Zorowitz et al. (2021) found that the symptom severity of anxiety, depression, mania, anhedonia, and worry were all higher in a group of online workers identified as careless/inattentive responders. Crucially, correlations between self‐report symptoms and cognitive performance reduced successively with the addition of each quality check measure. Why would inattentive responding introduce systemic bias? The authors show that the answer lies in mental health measures that are nonnormally distributed in the general population (i.e., positively skewed). In these cases, random responding to questionnaire items produces scores that are higher than the true mean. Coupled with the fact that random responding tends to yield poorer performance on measures of cognitive ability, this introduces systematic bias—specifically negative correlations between symptom severity and task performance (Zorowitz et al., 2021).

## 4 THE CHALLENGE OF QUALITY CONTROL IN AMT

These findings are stark and may rightfully prompt researchers to adopt a greater number of checks to protect the integrity of their online data. The unfortunate reality is that these protective methods are becoming less effective as the sophistication of “bots” (semi/fully automated scripts that mimic human behavior) increases over time, evinced by the data of Burnette et al. (2021). In our experience, an effective method of restricting the participation of negligent respondents is to require all entrants to consume information on one page (e.g., instructions about a cognitive task), and then later pass a test based *on comprehension* (rather than straight regurgitation) of that information. While this approach yields dramatic improvements in data quality and reduces experimenter costs as subjects are screened for attention prior to study entry, it too has issues. It may introduce sampling bias because some clinical populations are associated with frequent lapses of attention and reduced motivation, introducing a trade‐off between the removal of potentially fraudulent respondents and screening out real patients that we want to be represented in research (Chmielewski & Kucker, 2019). Additionally, genuine workers who either make honest mistakes or fail to comprehend the instructions you provide will not be allowed to enter a study, having already committed time they could have allocated to another online task. This creates an opportunity cost that has ethical implications. This also dovetails with issues around informed consent that have been largely unaddressed in online research. This sort of comprehension check could be used in future studies to assess cognitive capacity prior to taking informed consent. If so, care needs to be taken to highlight unpaid prestudy requirements upfront, and ensure those implemented are fair, brief, and clear.

Ultimately though, we do not believe that an ever‐increasing list of attention and quality checks is the solution to data quality issues in online research. Perhaps the single biggest change we can make is to appreciate that the incentives of workers on AMT are fundamentally not aligned with those of the researcher (at least, in most cases). The AMT workforce is not a group of psychology enthusiasts sitting at the other end of a screen wanting to kill time and chip in for the sake of research. There are many flavors of workers on AMT, a small number may have bad intentions and develop bots to exploit weaknesses in study designs, but a great many come from low‐income settings trying to earn a living wage from a finite resource—their time. If we are to continue to conduct research on this platform, we need to accept that not all study designs are appropriate. It appears poor quality responses are more pronounced in survey‐based designs where (i) misrepresentation is sometimes encouraged by our inclusion criteria, (ii) thoughtful reflection and deliberation is not incentivized and (iii) quick completion times are inadvertently rewarded (due to opportunity cost of time). Moving forward, we need to be more aware of the motivations of our participants in online research. Studies should be designed so that participants derive value from engaging carefully and earnestly. While this can be challenging for surveys, for cognitive testing this might include integrating bonuses within our tasks that make honest engagement worthwhile. In online research we want humans to be on the other end of the screen, so we need to treat them as such. Experiments must be accurately described, paid fairly, and not so long and repetitive that workers feel dizzy by the end.

## 5 ONLINE RESEARCH IN A POST‐TURK ERA

For many AMT workers the financial pressure to rapidly complete as many tasks as possible will ultimately put a ceiling on data quality. With this in mind, we highlight an emerging role of “Citizen Science.” Citizen scientists are members of the public who donate their time to solve research problems that require lots of people to contribute in a small way, usually in partnership with academics, and without payment. Citizen scientists engage because they are interested in science or crucially, they resonate with the cause, in many cases having lived experience with a mental health condition. This reduces generalizability of findings but can also enrich a study for people to whom the questions are most relevant. In our experience, data quality is best when the motivations of researcher and participant are well‐aligned. For citizen science to succeed, researchers must invest resources in developing user‐friendly, stylized interfaces, adopt gamification and most importantly, share insights/outputs with those who donate their time. Smartphone apps are providing particularly elegant avenues for citizen engagement, where participation rates range from the tens of thousands to millions (Gillan & Rutledge, 2021). This method comes with its own sample biases of course; participants must be willing and able to provide their time without monetary compensation. All approaches, paid and unpaid, targeted and broad, have their limitations and is it crucial to recognize that generalizable research insights require convergent evidence across multiple studies using different approaches. For studies that target highly specific populations (e.g., transgender individuals or people starting mental health treatment), we have found hybrid approaches that use closed recruitment channels to identify eligible and mission‐aligned participants (e.g., community forums, mental health providers), can include compensation for time and emotional labor without sacrificing data quality. Taking this approach, we recently found data quality to be excellent in a large group of depressed individuals participating in longitudinal online paid research. The most common answer for what subjects liked most about the study was the opportunity for self‐reflection (23%), while just 3% cited the money they received.

## 6 CONCLUSION

Online research, in its various forms, flawed, complex, and ever evolving, is a powerful tool for research in brain health. It has not just dramatically scaled up our studies, that is, it allows us to conduct rich, repeated and ecologically valid research of a sort that is not practical in‐person (Gillan & Rutledge, 2021). This is important because online, remote, and smartphone‐based tools will be essential if we are to implement research findings in clinical practice in a scalable way. However, the reality is that careless, inattentive and even outright fraudulent responses in online‐based assessments are inevitable and will continue to rise if we do not adapt our study designs. Worryingly, not all studies are designed as rigorously as Burnette et al. (2021) and so, a major unanswered question is; to what extent has this already affected published research? There is a pressing need for the field to develop standardized protections and quality assurances for evaluating online studies, but this task is not straightforward. The inclusion of captchas, repeated items and inattention checks are already starting to show diminishing efficacy—this field requires energy, agility, self‐scrutiny, and when it does not work out, we need researchers that are willing to share their experiences, exactly as Burnette et al. (2021) have done.
